# Diffuse Idiopathic Skeletal Hyperostosis (DISH) With Cervical Myelopathy: A Case Report From Sudan

**DOI:** 10.1002/ccr3.73643

**Published:** 2026-09-28

**Authors:** Elham Abdalla, Mohammed Alfatih, Mohammed Alssir MohammedAhmed, Amel Siddig, Hassan Zeyada

**Affiliations:** ^1^ Department of Internal Medicine Bahri University Khartoum Sudan; ^2^ Alzaiem Alazhari University Khartoum Sudan; ^3^ Faculty of Medicine Ahfad University for Women Khartoum Sudan; ^4^ Omdurman Teaching Hospital Khartoum Sudan; ^5^ Khartoum North Teaching Hospital Khartoum Sudan

**Keywords:** case report, cervical myelopathy, diffuse idiopathic skeletal hyperostosis, ossification of ligaments, spinal canal stenosis

## Abstract

Diffuse idiopathic skeletal hyperostosis (DISH) is a non‐inflammatory condition characterized by ossification of spinal ligaments, commonly affecting elderly individuals. While often asymptomatic, it may present severe neurological deficits due to spinal cord compression. We report what is believed to be the first documented case of DISH presenting with cervical myelopathy in Sudan. A 73‐year‐old diabetic man presented with progressive weakness in all limbs, accompanied by neck and back pain. Neurological examination revealed spastic quadriparesis, sensory loss to proprioception and vibration, and signs consistent with cervical myelopathy. No bowel or bladder involvement was reported. Imaging studies including CT and MRI of the spine demonstrated ossification of the anterior and posterior longitudinal ligaments throughout the cervical region, with severe spinal canal stenosis at C5–C7. Additional lumbar stenosis due to ligamentum flavum hypertrophy was also noted. A diagnosis of DISH with cervical myelopathy and lumbar stenosis was made. Surgical decompression was planned; however, the patient died unexpectedly before the planned intervention. DISH with cervical myelopathy is rarely encountered in clinical practice and has traditionally been considered more prevalent among Asian populations; however, we report what is, to our knowledge, the first documented case of DISH with cervical myelopathy in Sudan. This case report underscores the importance of early recognition of DISH in settings where it is rarely reported, in order to improve clinical outcomes. Furthermore, this case report highlights the need for further epidemiological and clinical studies in the country to assess the prevalence, risk factors, and management outcomes associated with this underrecognized rheumatologic disorder.

## Key Clinical Message

Diffuse Idiopathic Skeletal Hyperostosis (DISH) can lead to severe cervical myelopathy due to extensive ligament ossification, even in populations where it is rarely reported. Early recognition with appropriate imaging, including cervical MRI, is crucial to prevent irreversible neurological deficits and improve patient outcomes.

**Abbreviations**
CT: computed tomography
DISH: diffuse idiopathic skeletal hyperostosis
MRI: magnetic resonance imagin

## 1 Introduction

Diffuse Idiopathic Skeletal Hyperostosis (DISH), also known as Forestier's disease or spondylitis ossificans ligamentosa, was originally reported by Forestier and Rotes‐Querol in 1950 [1]. DISH is a condition that occurs primarily in the elderly population, affecting 32.1% of men and 16.9% of women aged 80 and above. It is also more common in the Asian population, particularly in Japan, where prevalence rates range from 1.9% to 4.3% [2]. DISH is a condition characterized by the ossification of ligaments and entheses at their attachment points to bones, primarily in the spine and extraspinal areas [3]. The most affected ligaments include the anterior and posterior longitudinal ligaments and vertebral arch ligaments [3].

The clinical presentation of DISH is variable. Some cases can be completely asymptomatic, whereas others can complain of painful stiffness, limited mobility, spinal deformity, and, in some cases present with myelopathy, which is typically caused by the ossification of the posterior longitudinal ligament [1, 4]. Furthermore, dysphagia, dyspnea, and stridor from pharyngoesophageal and tracheal compression have been documented in the literature [4, 5, 6]. Although the exact cause of DISH remains unknown, it is strongly associated with increasing age, obesity, and type 2 diabetes. These conditions increase the risk by promoting the production of growth factors such as insulin‐like growth factor 1, as well as inflammatory mediators like IL‐1 and IL‐6, which stimulate osteoblast proliferation and lead to bone deposition through the receptor activator of the nuclear factor kappa‐B ligand pathway [1]. Additionally, there is some evidence of a genetic predisposition to posterior longitudinal ligament ossification that has been identified on chromosome 6p in DISH cases [4]. Other factors reported to contribute to the development of DISH include a protein‐rich diet and sedentary work [4].

Herein, we present a rare case of a patient with DISH who developed cervical myelopathy. The cause was ossification of the anterior longitudinal ligaments throughout the entire cervical region, as well as ossification of the posterior longitudinal ligament causing spinal canal stenosis, particularly at the C5, C6, and C7 levels. The neurological aspects of DISH are reviewed herein.

## 2 Case Presentation

### 2.1 Case History/Examination

A 73‐year‐old male presented with progressive weakness in both upper and lower limbs over a two‐month period, accompanied by neck and back pain. The patient had a 14‐year history of diabetes, managed with oral hypoglycemic medications. The patient denied any history of trauma.

On examination, the patient was wheelchair‐bound. A neurological assessment revealed spastic quadriparesis with brisk supinator, knee, and ankle reflexes, an extensor plantar response, a positive myelopathy hand sign, and pseudoathetosis (writhing finger movements with eyes closed). There was also a sensory loss to proprioception and vibration. No bowel or bladder dysfunction was noted.

### 2.2 Investigations

Routine laboratory and serological investigations were all within normal limits. Imaging studies including a cervical spine CT (Figure 1), a cervical spine MRI (Figure 2), and an MRI of the lumbosacral spine (Figure 3) were performed. The cervical CT (Figure 1) showed straightening of the cervical spine with loss of lordosis, ossification of the anterior longitudinal ligament throughout the entire cervical region, and ossification of the posterior longitudinal ligament causing spinal canal stenosis, particularly at the C5, C6, and C7 levels. No craniocervical pathology was detected. The cervical MRI (Figure 2), although of suboptimal image quality, demonstrated multilevel flowing anterior ossification consistent with DISH, with relatively preserved intervertebral disc heights. Posterior element hypertrophy and posterior longitudinal ligament ossification were also evident, causing significant spinal canal narrowing. At the mid‐cervical levels (C5–C7), clear spinal cord compression was observed, with marked reduction of the surrounding cerebrospinal fluid space. Subtle intramedullary T2 signal changes were noted at the levels of maximum compression, suggesting underlying compressive myelopathy (Figure 2). In addition, the MRI of the lumbosacral spine (Figure 3) showed an L3/L4 diffuse disc bulge with ligamentum flavum hypertrophy, indenting the thecal sac and causing significant bilateral nerve root impingement, leading to acquired canal stenosis. The L5/S1 level exhibited a mild diffuse disc bulge (Figure 3) with a T2 hyperintense disc signal, suggestive of early disc degeneration (Figure 2).

**FIGURE 1:**
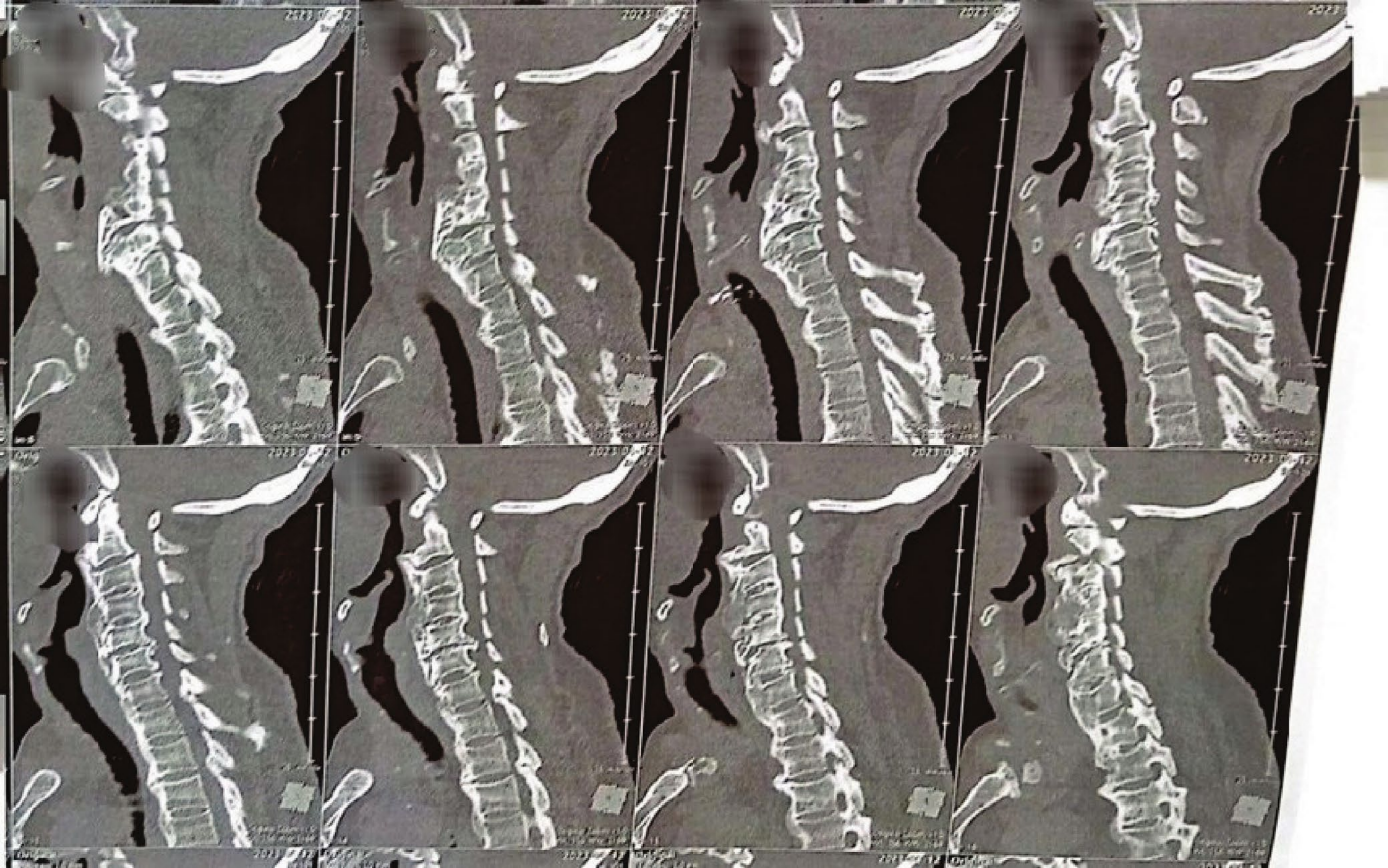
CT cervical spine (sagittal views). Showed Extensive ossification of the anterior longitudinal ligament throughout the entire cervical region. Ossification of the posterior longitudinal ligament is also evident within the spinal canal, contributing to significant canal narrowing, particularly at C5–C7. Loss of normal cervical lordosis is noted.

**FIGURE 2:**
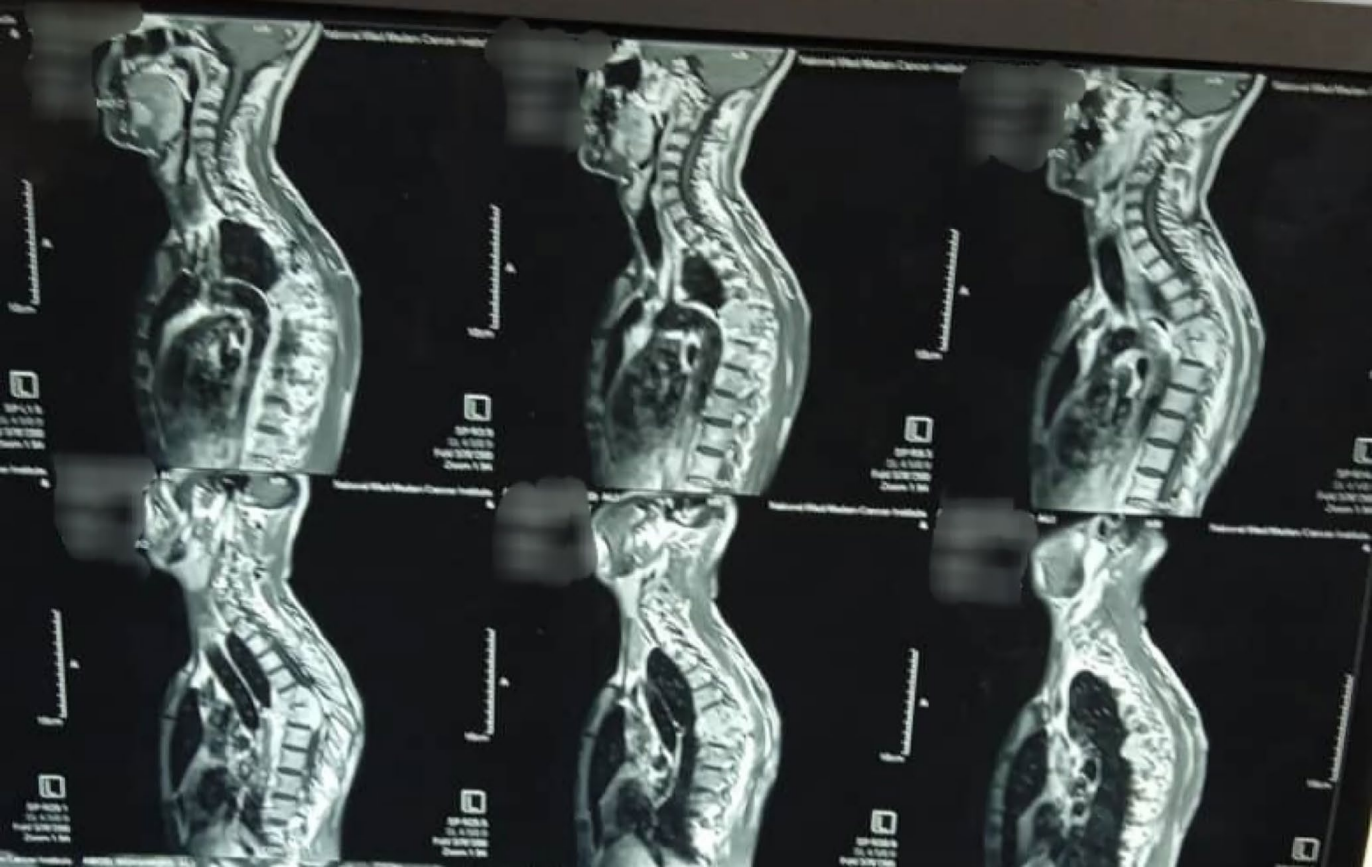
MRI cervical spine (sagittal views). Showed findings consistent with DISH, with relatively preserved intervertebral disc heights. Clear spinal cord compression, with marked reduction of the surrounding cerebrospinal fluid space, is also noted.

**FIGURE 3:**
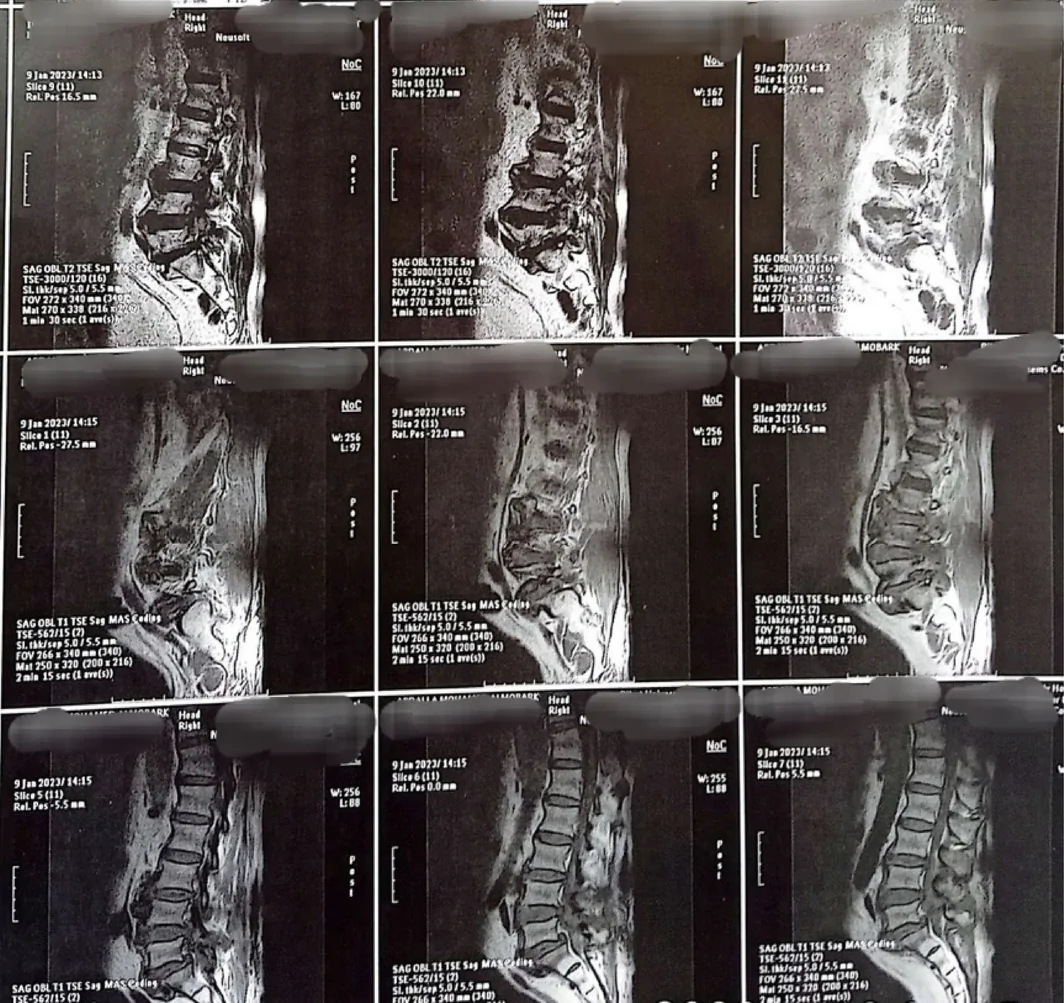
MRI Lumbosacral spine (sagittal views). Showed an L3/L4 diffuse disc bulge with ligamentum flavum hypertrophy. The L5/S1 level exhibited a mild diffuse disc bulge.

### 2.3 Differential Diagnosis

The patient showed no serological or radiological evidence of alternative rheumatologic conditions such as ankylosing spondylitis or rheumatoid arthritis. Based on clinical and radiological findings, a final diagnosis of DISH with compressive cervical myelopathy and lumbar spine stenosis secondary to ossification of the anterior and posterior longitudinal ligaments and ligamentum flavum was made.

### 2.4 Treatment

The patient was initially managed with analgesics and physical therapy. Subsequently, he was referred to the surgery department, where cervical decompression and fixation surgery was planned. Unfortunately, due to the ongoing conflict in the country, access to healthcare services became nearly impossible. Nevertheless, the patient was educated about his condition and the importance of promptly reporting any new symptoms. Follow‐up was conducted via telephone. However, after 3 months, the patient died suddenly before the planned surgical intervention could be performed.

## 3 Discussion

This case report highlights a presentation of DISH with cervical myelopathy diagnosed at a relatively old age. The incidence and prevalence of DISH increase with age and only a limited number of studies have reported cases in individuals aged 50 years or younger [7]. However, the reported prevalence of DISH is extremely variable according to the population studied [8].

DISH shares clinical and radiological features with degenerative spondylosis and ankylosing spondylitis, and other degenerative diseases [8, 9]. To differentiate DISH from these conditions, Resnick and colleagues in the 1970s proposed the following criteria including “flowing” ossification extending over at least four adjacent vertebrae, relative preservation of intervertebral disc height appropriate for the patient's age, and no evidence of apophyseal joint ankylosis or sacroiliac inflammatory changes [10]. The patient in this case report met all these criteria.

In a similar context, degenerative spondylosis involves disc degeneration, osteophyte formation, and disc space narrowing. In contrast, DISH primarily affects ligaments, displaying flowing calcification along the anterior and posterior longitudinal ligaments without significant disc degeneration. While DISH presents with stiffness and limited mobility, spondylosis often causes pain and radiculopathy due to disc herniation. Furthermore, myelopathy due to posterior longitudinal ligament ossification is a hallmark of DISH but is rare in spondylosis [2, 8, 9]. Conversely, ankylosing spondylitis is a chronic inflammatory disorder typically affecting younger individuals and characterized by sacroiliitis, the formation of a “bamboo spine,” and systemic inflammatory features. In contrast, DISH is a non‐inflammatory condition primarily seen in older adults and notably lacks both inflammatory features and sacroiliac joint involvement [8].

Radiological findings further distinguish these conditions. DISH presents with flowing ossification and preserves disc height, whereas ankylosing spondylitis features syndesmophytes and sacroiliac joint fusion. Recognizing these distinctions is critical for accurate diagnosis, as myelopathy in DISH requires timely intervention to prevent further neurological deterioration [8]. Although cases of DISH with cervical myelopathy have been reported in the literature, to the best of our knowledge, this is the first case report from Sudan.

A strength of this study is that the patient first presented to our institution with neurological symptoms and signs. Based on history taking, physical examination, and imaging studies including CT and MRI of the cervical and lumbosacral spine, the patient was diagnosed with DISH. To the best of our knowledge, this is the first reported case from Sudan. One limitation of this study is that, although surgical intervention was planned, follow‐up was conducted only by phone due to the ongoing conflict in the country. After 3 months of follow‐up, the patient died suddenly before the planned surgical intervention could be performed.

## 4 Conclusion

DISH has traditionally been considered more prevalent among Asian populations; however, we report what is, to our knowledge, the first documented case of DISH with cervical myelopathy in Sudan. While many cases of DISH remain asymptomatic, the condition can lead to catastrophic spinal cord injuries with a high risk of mortality, as demonstrated in this case report. DISH can no longer be viewed as a disease exclusive to the Asian population; this case report underscores the importance of early recognition of DISH in settings where it is rarely reported, in order to improve clinical outcomes. Furthermore, epidemiological and clinical studies are warranted in Sudan to assess the prevalence, risk factors, and management outcomes associated with this underrecognized rheumatologic disorder.

## Author Contributions

**Elham Abdalla:** conceptualization, data curation, project administration, supervision, writing – original draft, writing – review and editing. **Mohammed Alfatih:** conceptualization, data curation, investigation, methodology, project administration, writing – original draft, writing – review and editing. **Mohammed Alssir MohammedAhmed:** investigation, methodology, writing – original draft, writing – review and editing. **Amel Siddig:** data curation, investigation, methodology, writing – original draft, writing – review and editing. **Hassan Zeyada:** conceptualization, data curation, methodology, project administration, supervision, writing – original draft, writing – review and editing.

## Funding

The authors have nothing to report.

## Consent

Written informed consent was obtained from the patient to publication of this report and any accompanying images in accordance with the journal's patient consent policy.

## Conflicts of Interest

The authors declare no conflicts of interest.

## Data Availability

The data that support the findings of this study are available from the corresponding author upon request.
